# MicroRNAs as Biomarkers for Predicting Complications following Aneurysmal Subarachnoid Hemorrhage

**DOI:** 10.3390/ijms22179492

**Published:** 2021-08-31

**Authors:** Wang-Xia Wang, Joe E. Springer, Kevin W. Hatton

**Affiliations:** 1 Sanders-Brown Center on Aging, Spinal Cord and Brain Injury Research Center, and the Pathology & Laboratory Medicine, University of Kentucky, Lexington, KY 40536, USA; 2 Spinal Cord and Brain Injury Research Center, and the Department of Neuroscience, University of Kentucky, Lexington, KY 40536, USA; jspring@uky.edu; 3 Department of Anesthesiology Critical Care Medicine, University of Kentucky, Lexington, KY 40536, USA; kevin.hatton@uky.edu

**Keywords:** aneurysmal subarachnoid hemorrhage, delayed cerebral vasospasm, delay cerebral ischemia, biomarker, microRNA, biofluid

## Abstract

Aneurysmal subarachnoid hemorrhage (aSAH) is a high mortality hemorrhagic stroke that affects nearly 30,000 patients annually in the United States. Approximately 30% of aSAH patients die during initial hospitalization and those who survive often carry poor prognosis with one in five having permanent physical and/or cognitive disabilities. The poor outcome of aSAH can be the result of the initial catastrophic event or due to the many acute or delayed neurological complications, such as cerebral ischemia, hydrocephalus, and re-bleeding. Unfortunately, no effective biomarker exists to predict or diagnose these complications at a clinically relevant time point when neurologic injury can be effectively treated and managed. Recently, a number of studies have demonstrated that microRNAs (miRNAs) in extracellular biofluids are highly associated with aSAH and complications. Here we provide an overview of the current research on relevant human studies examining the correlation between miRNAs and aSAH complications and discuss the potential application of using miRNAs as biomarkers in aSAH management.

## 1. Introduction

Aneurysmal subarachnoid hemorrhage (aSAH) occurs when an intracranial arterial aneurysm ruptures, resulting in the release of oxygenated blood into the subarachnoid space where it mixes with cerebrospinal fluid (CSF) [1]. In some cases, this blood may also penetrate into the brain parenchyma or into the ventricular system, causing additional injury [1,2]. aSAH accounts for approximately 5% of all strokes and affects relatively younger individuals, compared to other types of stroke, with about 56% of the patients being less than 60 years of age [3]. aSAH typically presents with a thunderclap headache that is frequently described as the “worst headache of my life” by awake patients. On computed tomography (CT) examination, there is classic high-attenuating filling of the normally dark subarachnoid space, including in the cisterns and brain sulci. The ruptured aneurysm is sometimes seen on special CT angiography or, more commonly, during diagnostic digital subtraction angiography (DSA) procedures.

aSAH results in significant morbidity and mortality, resulting in death before or during initial hospitalization in nearly 30% of aSAH patients, and in those patients who do survive, almost 25% are at high risk of suffering secondary neurologic injuries resulting in chronic, lifelong neurologic and functional disabilities [4,5]. Aside from the severe clinical impact of the initial aneurysmal rupture, the poor prognosis can be caused by several neurological complications such as delayed brain injury (DBI) [6,7,8], hydrocephalus [9,10], and re-bleeding. 

DBI is the most significant cause of long-term poor prognosis and disability. DBI is a heterogeneous term for several complex clinical phenomena, including delayed cerebral vasospasm (DCV) and delayed cerebral ischemia (DCI). DBI events occur predominantly 4 to 10 days after aneurysm rupture in 30–70% of aSAH patients. Unfortunately, the pathophysiology of DBI is poorly understood and effective treatment paradigms have not translated well from the bench to the bedside. 

DCI is consistently associated with highly negative clinical outcomes and permanent disability. DCI occurs when there is an acute deterioration in neurologic status that cannot be explained by the initial aneurysm rupture or its direct consequences. Various mechanisms such as blood-brain barrier (BBB) disruption, inflammatory responses, microcirculatory dysfunction, microthrombosis, cortical spreading depression, and compromised cerebral blood flow autoregulation have all been proposed [11]. The diagnosis of DCI is often based on exclusion of other diagnoses such as infection, hypotension, hydrocephalus, etc. [12], and is especially difficult to diagnose in patients who remain comatose from the initial aneurysm rupture or who require sedation [13]. Understanding the exact mechanisms that lead to DCI will aid the discovery of effective biomarkers, as well as the development of future treatment strategies.

DCV is also a major cause of DBI [8,14,15] and occurs when intracranial vessels spasm, resulting in decreased blood flow to brain tissues distal to the site of spasm. Historically, DCV was thought to lead directly to DCI if brain tissue perfusion was not rapidly restored. DCV and DCI may be seen together in the same patients or separately without evidence of the other [16,17,18]. In addition, a systematic review and meta-analysis of 14 studies prior to 2009 showed that pharmaceutical treatments focused on reducing DCV did not significantly improve clinical outcome [18]. However, this discordance could be a result of study variations in pharmacological compounds, methodology, sample size, and clinical outcome measures. On the other hand, a more recent meta-data analysis of 17 studies on 2870 aSAH patients reveals that evidence of cerebral vasospasm using transcranial doppler is predictive of DCI with a high degree of sensitivity (90%) [19]. This suggests that DCV prediction may be an important proxy for DCI, perhaps especially prior to its onset and in high-grade aSAH patients in which the neurologic examination is obscured by acute injury and/or sedation and the prevention of further secondary brain injury is critical for survival.

Early diagnosis and treatment appear to be key to the clinical management of DBI, however, due to the multifactorial nature of DBI, there is no reliable scoring system or biomarker to predict DBI occurrence. Currently, diagnosis of these complications relies on imaging and a “wait and see” approach, which often miss identifying the optimal treatment times. 

MicroRNAs (miRNAs) play key roles in posttranscriptional gene regulation in normal biological and pathophysiological processes that underline many human disorders including neurological diseases [20,21,22,23,24,25,26,27]. MiRNAs are highly sensitive to cellular stimuli and pathophysiological conditions and are directly involved in the regulation of several key pathophysiological events following stroke including apoptosis, neuroinflammation, oxidative stress, brain edema, neurogenesis, and angiogenesis [28,29,30,31,32,33]. It is thus conceivable that alterations of miRNAs in biofluids might accurately reflect ongoing acute pathophysiological events. Indeed, the levels of biofluid miRNAs have been associated with both ischemic and hemorrhage stroke, including subarachnoid hemorrhage [34,35,36,37]. A recent metadata analysis supports the notion that different biofluid miRNA fingerprints are associated with the clinical progression of aSAH and can serve as potential biomarkers in aSAH management [38]. In this review, we summarize recent research investigating the association between biofluid miRNAs and aSAH complications, focusing primarily on potential miRNA indicators of DCV and DCI outcomes. We also discuss the pros and cons of the different types of specimens, collection times, and analysis methodologies from the perspective of clinical applications using miRNA biomarkers for the management of aSAH.

## 2. MiRNA in Intracranial Aneurysmal (IA) Tissue and Subarachnoid Hemorrhage

Intracranial aneurysm (IA) is a cerebrovascular disorder in which cerebral artery is pathologically weakened and ballooned. When ruptured, intracranial aneurysm causes life-threatening aSAH. It is well documented that miRNAs play fundamental roles in vascular integrity and vascular function [39,40,41,42,43]. For example, studies have reported that a deficiency in the miRNA biogenesis machinery protein, DICER, in vascular smooth muscle cells results in defective blood vessel formation and embryonic lethality [43]. In addition, miRNAs are aberrantly expressed in the vascular walls following injury [42]. Several studies have confirmed that miRNAs are dysregulated in intracranial aneurysms (Table 1) [30,44,45,46,47,48]. In 2014, Liu et al. reported that over 150 miRNAs were differentially expressed in aneurysmal arteries compared to normal arteries [30], demonstrating both patterns of up- and down-regulation of various miRNAs. However, a previously published study [45] identified much fewer IA-associated miRNAs (18 miRNAs) with all being down-regulated. While the reason for the differences between these two studies is not apparent, it is important to note that the sites of sampling for the control groups between the studies were significantly different. Specifically, Liu et al. used the extracranial superficial temporal artery; whereas, Jiang et al. used the intracranial middle meningeal artery. In addition, a recent study by Supriya et al., employed intercostal artery as the sampling site for control specimens and identified 70 dysregulated miRNAs in aSAH [47]. Unlike the above-mentioned studies that analyzed ruptured aneurysmal tissue, Bekelis et al. utilized unruptured aneurysms to investigate miRNAs as well as protein coding genes in the same specimen. These authors detected a number of differentially expressed miRNAs and also significantly altered mRNAs/genes that were reversely correlated with the differential miRNA expression patterns [44]. Wei and colleagues took a similar approach by analyzing an existing dataset (GSE54083) originally generated by Nakaoka et al. [49] and identified 12 IA-associated miRNAs that were differentially expressed [48]. However, these authors did not report whether a reverse correlation exists between the levels of the 12 miRNAs and the altered gene expression levels. 

The majority of identified IA-associated miRNAs include those that are enriched or implicated in endothelial and vascular smooth muscle function (miR-23b, miR-143, miR-145, miR-9, miR-1, miR-10, miR-17, miR-24-1, and the let-7 family), as well as miRNAs involved in inflammatory responses and immunity (miR-125b, miR-155, miR-21, and miR-146a). It is worth mentioning that many of these differentially expressed miRNAs (including miR-143, miR-145, miR-21, miR-26, miR-29a/b, miR-146a, miR-155, miR-133a, miR-133b, and let-7 family) are also altered in abdominal aortic aneurysmal tissues [50,51,52,53] suggesting a common mechanism. Interestingly, the levels of several miRNAs (e.g., miR-143, miR-145, and miR-23b) were found to be consistently down-regulated among the studies described above [30,45,47,54]. For example, miR-23b was reduced in IA tissues and was reported to target phosphatase and tensin homolog (PTEN) [55], a key regulator of proliferation, differentiation, and cytokine production during pathological vascular remodeling in smooth muscle cells [56]. MiR-143 and miR-145 play an important role in controlling vascular smooth muscle phenotype by maintaining or inhibiting differentiation [28,57] and were found to be significantly down-regulated in IA tissues and plasma [30,44,45,47,54,57]. Consistent with a decrease in miR-143 and miR-145, their predicted target genes, including those involved in extracellular matrix remodeling, collagen synthesis, and metabolism (ADAMTS2, COL1A1, COL5A1, and COL5A2), were significantly up-regulated in the same tissues [44]. In addition, miR-143 and miR-145 were found to target Krüppel-like factor 5 (KLF5) [54], a transcription factor that plays an essential role in vascular remodeling [58] and is strongly induced in activated smooth muscle cells under pathological conditions [59]. However, rodent studies seem to contradict the observations from the human studies. Upregulation of miR-143 was observed in cerebral arteries after aSAH in a rat model [31], and another study found that miR-143 and miR-145 deficiency significantly reduces atherosclerosis in mice [60]. Further investigations will be required to clarify the role of these miRNAs in aSAH. Another miRNA regulates endothelial and vascular smooth muscle function is miR-9. The expression of miR-9 was found to increase in IA tissues and negatively regulated MYOCD (Myocardin) [46], a co-activator of serum response factor (SRF), which play a crucial role in cardiogenesis and differentiation of smooth muscle cell lineage and is an essential player in controlling vessel contraction [61].

miRNAs are recognized as powerful regulators of central nervous system (CNS) inflammatory responses [32], including inflammation that occurs following ischemic stroke and aSAH [29,62,63,64]. For example, miR-125b was shown to be reduced in IA tissues [30,47,48], which may target nitric oxide synthase 1 (NOS1) and contribute to macrophage-mediated vascular smooth muscle cell apoptosis [65]. MiR-155 is a major inflammatory responsive miRNA that is immune cell-specific and highly inducible [32]. MiR-155 was found to be up-regulated in unruptured IA tissues compared to ruptured tissues and matrix metalloproteinase-2 was identified as miR-155 target in a report by Yang et al. [66]. However, miR-155, together with miR-146a, miR-223, and miR-124a, were not significantly changed according to a previous study [30]. Because the two groups used different vascular tissues in their studies, the results may not be comparable. In addition, it is also unclear whether the up-regulation of miR-155 was a result of immune cells that infiltrated the IA tissue. MiR-21 is a multi-faceted miRNA that participates in the process of cellular proliferation, migration, and apoptosis in vascular tissues [67]. MiR-21 was found to be highly up-regulated in unruptured IA tissues [44] and was associated with multiple downregulated target genes such as poly(A) binding protein interacting protein 2B.

Taken together, these studies demonstrate a strong involvement of miRNAs in the pathogenesis of IA and aSAH and provide a wealth of information for studying the mechanisms associated with these two vascular events. On the other hand, inconsistent findings are seen across studies, which are most likely due to limitations related to the heterogeneity of the specimens examined, study design, and small sample sizes.

## 3. Alteration of Biofluid miRNAs Associated with aSAH and the Complications

The use of biofluid miRNA biomarkers in aSAH has been explored in a number of recent studies (Table 2). Encouraging data confirm that dysregulated miRNA expression is associated with aSAH, DCV, and DCI, and that changes can be observed in biofluids and circulating blood cells [62,63,68,69,70,71,72,73,74,75,76,77,78,79,80,81]. Importantly, altered miRNA expression levels in biofluids following aSAH are temporally dynamic and are most noticeable within the first 3 days post-ictus [62,69,71,75,77,81]. This period is critical in aSAH as it directly links the early, acute brain injuries to DBI, likely through the initiation of a cascade of secondary events such as oxidative stress, neuroinflammation, and apoptosis [82].

A number of miRNAs have emerged as consistent indicators of aSAH and related complications (Table 2). For example, based on the results of several observation studies, many different miRNAs are highly elevated in CSF of aSAH patients compared to controls [68,69,71,79,81]. In particular, elevated levels of let-7b-5p, miR-19b-3p, miR-125-5p, miR-221-3p, miR-21-5p, and miR-27a-3p in CSF are all highly predictive of DCV [68,69,79,81].

The alterations of plasma miRNAs in aSAH are less robust, and are substantially different from that of CSF. Several plasma miRNAs collected at day 3 post ictus, including let-7a-5p, miR-146a-5p, miR-204-5p, miR-221-3p, miR-23a-3p, and miR-497-5p, showed strong DCV predictive potential with AUCs ranging from 0.84 to 0.98 [81]. In addition, elevated miR-3177 was showed to associate with DCV [76]. However, no miRNA was found to associated with DCV in blood samples collected after day 7 [63], suggesting that earlier sampling time points may be more optimal. Likewise, differentially expressed plasma miRNAs collected at day 1 following aneurysm rupture can effectively distinguish aSAH patients from controls [74,80], but this differential expression pattern is lost when using the plasma collected at day 3 or later [62,72,81]. These studies strongly suggest that the timing of sample collection is critical to capture the biomarker signatures associated with aSAH and associated complications.

Changes in miRNAs following aSAH have been implicated in prognosis in several studies. Elevated CSF miR-9-3p and miR-9-5p are associated with poor functional outcome [69], and serum miR-502-5p and miR-1297 may help predict neurological outcome [72,77,78]. Unfortunately, to date no miRNA has been identified that significantly associates with DCI [68,69].

## 4. Current Limitation of Using miRNAs as Biomarkers for aSAH and Its Complications

The current management of aSAH and its short and long-term complications relies solely on imaging and neurological examination [12]. However, this management regime depends heavily on a “wait and see” approach which can result in therapies that ultimately miss the optimal treatment window. In addition, imaging and the neurological examination may not effectively identify pathologic and subtle neurological changes prior to the onset of a serious complication. These challenges warrant a need for biomarkers that are able to significantly improve our ability to predict, diagnose, and monitor clinical deterioration. Although protein and metabolite biomarkers involving neuronal, vascular, angiogenic, coagulation, and inflammatory response associated with aSAH and its complications have been analyzed, the sensitivity and specificity are of major concerns for the utility of these potential biomarkers [85,86,87]. In particular, several protein markers, such as glial fibrillary acidic protein, neuro-specific enolase, and S100 calcium binding protein B, a protein involves in BBB dysfunction and brain lesion [88], are also present in stroke and traumatic brain injury [89,90]. Compared to protein and metabolite biomarkers, the advantages of using miRNAs as biomarkers for aSAH and DBI include tissue/cell type, pathophysiological specificity, and stability in biofluids [91], as well as ease of detection [35,92,93,94].

The complex and heterogeneity of aSAH pathophysiology is a major challenge in interpreting the functional involvement of miRNAs as a biomarker for DBI and poor neurologic outcomes. Unfortunately, the use of miRNA as a biomarker in aSAH is also currently limited by significant variability in the published literature in the approaches used, including differences in sample size (between 4 and 129 cases), types of biofluid used (whole blood, CSF, serum, plasma, exosome etc), collection time points (ranging from 1 to 14 days to 2 years), different methodologies for miRNA analysis (microarray, NGS, RT-qPCR), as well as data normalizations. These pre-analytical, analytical, and post-analytical factors need to be optimized in order to achieve reproducibility during biomarker discovery and must be established before miRNA biomarkers can be applied in clinical practice.

### 4.1. Type of Biofluids

Several types of biofluids including plasma, serum, whole blood, and CSF have been used to identify valid aSAH biomarkers. Blood (plasma, serum, and whole blood) is the preferred source for biomarkers in many different diseases and conditions. However, since blood circulates freely within multiple organ systems, its constituents (proteins, metabolites, miRNAs etc.) reflect a more global, systemic state, and there can be a high degree of non-specificity. In addition, many CNS-related molecules are not able to cross the BBB into the blood stream. These factors make blood as a source of biofluid miRNAs difficult to interpret. The utility of miRNA analysis in blood may be enhanced by isolating specific types of exosomes, as they can cross the BBB and may reflect cellular origin and cell/tissue-specific pathological processes. At this time, the utilization of biofluid exosomes remains challenging due to a difficult and lengthy isolation procedure, a varied and low yield, and a lack of specificity for exosome markers [95]. 

CSF, on the other hand, is highly specific to CNS injuries and conditions, although more invasive and difficult to sample compared to blood. However, the high frequency of external ventricular drain placement (>50% of aSAH patients) to treat hydrocephalus and elevated intracranial pressure [96] allows for access to collection of CSF across multiple time points. MiRNA alterations in CSF are vastly different from that seen in blood, and the changes are of a much greater scale. CSF miRNAs are potentially better candidates as aSAH complication biomarkers and may be more effective in predicting DCV and functional outcomes [68,69,79,81]. On the other hand, no miRNAs have been identified to consistently associate with DCI in aSAH. This is largely because of the heterogeneity nature of DCI and the lack of understanding of the underlying pathophysiological pathways. Future investigation will need to consider searching additional miRNAs or groups of miRNAs and other molecules in both CSF and blood to improve the chances of identifying DCI-specific biomarker(s).

### 4.2. Specimen Sampling Time

Another caveat with the existing studies is the significant variability of specimen collection times. Because miRNA responses are associated with a certain biological or pathological event, the alterations of miRNAs are time-dependent. This is supported by current studies showing that biofluid miRNAs are temporally dynamic following aSAH (Table 2). Therefore, it is necessary to determine an optimal sampling time that reflects the underlying pathology and provides the greatest degree of biomarker differentiation. Depending on the design and purpose of the study, the sampling times in the published literature ranges from hours to months to years. For assessing general outcomes and prognosis, the sampling times may have a wider window during the course of aSAH. However, when assessing relevant biomarkers for DBI, it is important to sample at early times (i.e., prior to day 4 following aneurysm ruptured) to ensure that the biomarker is predictive and may inform clinical decision-making or therapeutic options before non-reversible complications arise. On the other hand, one should also be cautious about using samples collected at very early time point, e.g., Day 1 following rupture. The presence of and changes in miRNAs in these very early samples may be released directly from injured or dead cells, which may not necessarily be reflective of DBI potential.

### 4.3. Method of miRNA Detection and Quantification

A significant challenge to quantifying miRNAs is their relatively low level in cell-free biofluids. Several reports have utilized very time-consuming and costly analytical methodologies (such as next-generation sequencing and microarray), which are appropriate for biomarker discovery but not suitable for clinical practice, especially when dealing with an aSAH complication and clinical decisions need to be made relatively quickly. Commonly used miRNA analysis platforms include RNA-Seq (requires higher quantity of RNA/more expensive/lengthy procedure), microarray (requires higher quantity of RNA/less sensitive/more expensive/lengthy procedure), Nanostring (fast-turn around/able to use poor quality RNA/less specific/requires special equipment), and qPCR (Table 3). Among these platforms, qPCR stands out for its sensitivity, specificity, cost-effectiveness, and turn-around time, all of which are important considerations for future translational applications.

### 4.4. Data Normalization and Analysis

Different methods of data normalization can profoundly affect the interpretation of data and the choice of normalization method is essential for miRNA biomarker discovery. The normalization methods used in the reviewed studies varied drastically. For example, Meeuwsen et al., [84] selected miR-15b-5p, miR-126-3p, miR-21-5p, miR-30c-5p, and miR-148b-3p as reference miRNAs. However, several of these miRNAs (such as miR-15b-5p and miR-21-5p) have been shown in other studies to be altered in aSAH specimens [71,81,83]. Bache et al. [69] specified that they were not able to apply sample mean normalization method in their dataset due to substantial variations in the mean of each CSF samples. The group eventually reported the findings based on non-normalized data. Compared to total RNAs, miRNA data normalization is challenging especially for extracellular biofluid miRNAs due to the lack of appropriate endogenous controls. Since miRNAs are highly cell/tissue-specific, it is not possible to identify an appropriate ‘house-keeping’ miRNA. The use of endogenous miRNAs as normalizers thus is highly empirical, which needs to be determined in a case-by-case biological context. Several algorithms such as geNorm [97], NormFinder [98], can be used to assist in identifying stable endogenous miRNAs as references. Exogenous synthetic oligonucleotides (such as cel-miR-39) are often used as ‘spike-in’ references. These ‘spike-in’ oligonucleotides may serve to monitor the efficacy of RNA isolation or reverse transcription and can be used to correct qPCR data for the variability related to these specific processes. However, spike-ins should not be used for normalization of biological data because they are unable to measure any endogenous variability within the specimens. Finally, global mean normalization [99] is useful in experiments in which there are a large number of miRNA assays. One suggestion for dealing with normalization challenges is to cross-test or combine different normalization methods and then validate the differential miRNAs when possible.

## 5. MiRNA Biomarker Panel for aSAH and Its Complications

**Figure 1:**
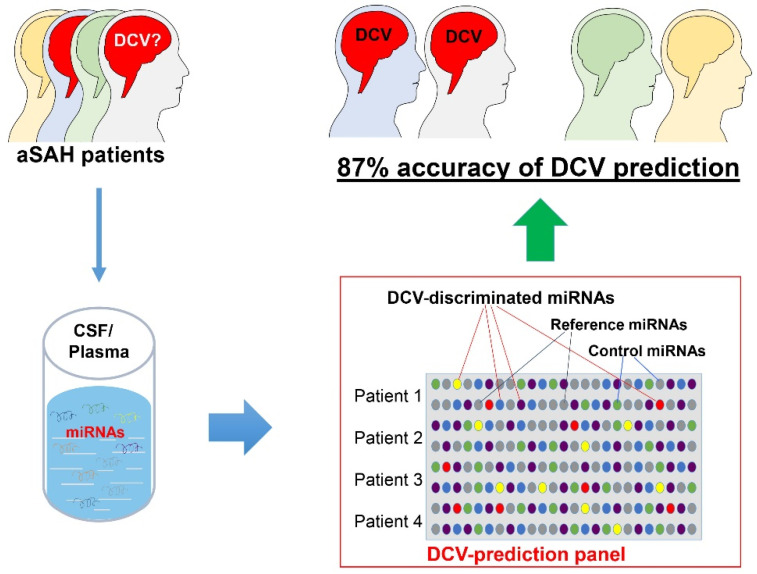
A highly predictive miRNA panel for DCV. The DCV miRNA predictive panel consists of miRNAs that are involved in multiple pathophysiological pathways related to vasculature and brain injury [81]. The panel also included reference and control miRNAs for data normalization. This panel was highly predictive of DCV using CSF specimens collected at 3 days following aneurysm ruptured.

The conventional process of biomarker discovery is based on either the understanding of molecular or biochemical mechanisms of the disease, or by large scale profiling and screening (aka. -omics). Both approaches have significant challenges in complex heterogeneous diseases and pathological conditions, including aSAH. Moreover, the altered expression of molecule(s) associated with a single pathway may not sufficiently reflect the underlying complexity of aSAH, and a panel of several molecules/biomarkers that are involved in multiple pathophysiological processes may be a much more effective strategy. This strategy is supported by the fact that the levels of multiple miRNAs rather than that of a single miRNA were significantly altered in IA tissue and aSAH complications (Table 1 and Table 2). We recently piloted a novel approach for developing a miRNA biomarker panel by creating a disease-focused miRNA panel [100]. We tested this strategy to study a set of miRNAs as biomarkers for DCV and achieved an 87% prediction accuracy in a small cohort (Figure 1) [81]. The selected miRNAs on the DCV panel are involved in multiple pathways such as endothelial and vasculature function, inflammatory/immune responses, BBB function, apoptosis, angiogenesis, autophagy, and mitochondria function. We also included potential endogenous normalizers in the panel. We then adapted the TaqMan qPCR technology to make the analysis fast and cost-effective. We believe such a biomarker panel can provide an effective tool to improve the sensitivity and specificity of risk assessment for aSAH and its associated complications.

## 6. Perspectives and Concluding Remarks

Biofluid miRNAs hold great potential as biomarkers for aSAH and its complications although the sensitivity, specificity, and reproducibility are not yet fully established. Future studies aimed at optimizing each stage of biomarker study from sample collection to biomarker assays and data analysis will certainly promote the development of miRNAs as aSAH biomarkers. Furthermore, targeting miRNAs to achieve therapeutic benefits in aSAH complication management is an attractive perspective and identification of miRNA biomarkers should provide insights into the mechanistic roles of miRNA in aSAH. In addition, manipulation of miRNA pathways in aSAH animal models may provide valuable information on the predictive value and therapeutic potential of miRNAs. Given the existing evidence and clear potential, it can be argued that the development of strategies utilizing biofluid miRNAs as aSAH biomarkers is warranted with the ultimate goal of being applied for the assessment, prediction, diagnosis, and monitoring of aSAH and its complications.

## Figures and Tables

**Table 1:** Differential miRNA expression in human IA tissues.

Author Reference/Year	Tissue	Detection	Major Differential Expressed miRNAs
Jiang et al. (2013) [45]	14 IA, 14 MMA	Agilent Microarray	miR-133b, miR-133a, miR-1, miR-143-3p, miR-145-3p, miR-145-5p, miR-455-5p, miR-143-5p, miR-23b-3p, miR-24-1, miR-29b, miR-29c
Liu et al. (2014) [30]	6 IA, 6 STA	Agilent Microarray	let-7 family, miR-17, miR-23b, miR-126, miR-24-1, miR-222, miR-143, miR-145, miR-1, miR-10a, miR-125b, miR-26a etc.
Bekelis et al. (2016) [44]	7 UIA, 10 STA	Affymetrix Microarray	miR-21, miR-143, miR-145, miR-1246, miR-6753, miR-6875-3p, miR-4685-3p, miR3195, miR-6068, miR-193b-5p etc.
Luo et al. (2016) [46]	13 IA, 10 MMA	qPCR	miR-9
Wei et al. (2018) [48]	8 IA, 10 STA	Agilent Microarray	miR-125a, miR-125b, miR-145, miR-146a, miR-21, and miR-214
Guo et al. (2018) [55]	32 UIA, 17 PA	qPCR	miR-23b-3p
Xu et al., 2018 [54]	30 IA, 30 STA	qPCR	miR-143, miR-145
Yang et al., 2019 [66]	48 IA, 46 UIA	qPCR	miR-155
Supriya et al., 2021 [47]	29 aSAH, 20 IcA	Exiqon Microarray, qPCR	miR-24-3p, miR-26b-5p, miR-27b-3p, miR-125b-5p, miR-143-3p, miR-145-5p, miR-193a-3p, miR-199a-5p, miR-365a-3p/365b-3p, and miR-497-5p

IA: intracranial aneurysm; UIA: unruptured IA; MMA: middle meningeal artery; STA: superfacial temporal artery; PA: pulmonary artery; IcA: intercostal artery.

**Table 2:** Major studies investigating biofluid miRNAs as biomarkers in aSAH and complications.

Author/Reference	Biofluids	Cohorts	Day of Collection	Detection Method	aSAH and the Complications	Differential miRNAs	Major Findings
Powers et al., 2016 [75]	CSF	4 DCI+, 4 DCI−	Day 3–12	Nanostring nCounter/qPCR	No comparisons provided	Time course of let-7b-5p, miR-92a-3p, miR-491-3p and more than 30 other miRNAs	CSF miRNAs are temporally differentiated abundance following aSAH over time
Stylli et al., 2017 [79]	CSF	10 DCV+, 9 DCV−, 4 Ctrl	Various, Day 1–18	Nanostring nCounter	aSAH vs. Ctrl DCV+ vs. DCV−	miR-204-5p, -223-3p, -451a, and more than 200 other miRNAs miR-27a-3p, -516a-5p, -566, and -1197	CSF miRNAs can distinguish aSAH from non-aSAH, and DCV from non-DCV patients.
Bache et al., 2017 [68]	CSF	27 aSAH (8 DCI+, 9 DCI−), 10 Ctrl	Day 5	TaqMan and LNA Exiqon qPCR	aSAH vs. Ctrl DCI+ vs. DCI− DCV+ vs. DCV− EBI	let-7b-5p, -125b-5p, -19b-3p and 66 other miRNAs miR-10b-3p, -21-5p, -132-3p, -146a-5p, -221-3p, -208a-3p, and other 5. miR-132-3p, -19b-3p, -210-3p, -221-3p, and -484 miR-9-3p	CSF miRNAs are altered following aSAH, and the changes are associated with DCI, DCV, and EBI.
Kikkawa et al., 2017 [71]	CSF Plasma exsosome	8 aSAH, 3 Ctrl	Day 1, 3, 5, 7, 9, 11, 13	Toray microarray (2 aSAH, 2 Ctrl, day 3), qPCR	aSAH vs. Ctrl	miR-16-5p, -19b-3p, miR-15a-5p, -15b-5p, -92b-3p, -29a-3p, and other 34 in CSF, and 13 in blood	MiR-15a expression was significantly increased in both CSF and plasma, with a peak around 3–5 days after aSAH
Bache et al., 2020 [69]	CSF	Discovery (63 aSAH, 11 Ctrl); Replicate (63 aSAH)	Day 1–10	Exiqon qPCR custom array	Poor outcome High WFNS DCI+ vs. DCI−	miR-9-3p, -9-5p, and 18 others miR-16-5p, -451a, and 11 others miR-130b-3p, -483-5p and 9 others (not significant after *p* value correction)	Elevated miR-9-3p and -9-5p are associated with a poor functional outcome. No miRNA was associated with DCI.
Wang et al., 2021 [81]	CSF, plasma	31 aSAH (13 DCV+, 18 DCV−), 8 Ctrl	Day 3, 7	TaqMan qPCR custom array	aSAH vs. Ctrl DCV+ vs. DCV−	CSF: let-7b-5p, miR-142-3p, miR-19b-3p, -20a-5p, and 24 others Plasma: let-7a-5p, miR-146a-5p, -204-5p, -221-3p, and 14 others CSF: let-7b-5p, miR-19b-3p, -20a-5p, 24-3p, -142-3p, and 37 other miRNAs Plasma: let-7a-5p, miR-146a-5p, -204-5p, -221-3p, and 25 others	A selection of specific brain and vascular injury related miRNAs are highly predictive of aSAH and DCV
Jin et al., 2013 [83]	Plasma *	6 IA w/daughter blebs, 6 IA no daughter bleb, 6 aSAH, 6 Ctrl	Un-specified	Microarray	IA w/daughter blebs vs. Ctrl IA no daughter bleb vs. Ctrl aSAH vs. Ctrl	miR-21, -22, let-7b, -miR-720, -92a and 63 others miR-21, -22, -1471, and 10 others miR-3945, -4314, -365, and 12 others	MiRNAs are differential expressed in plasma of patients bearing different type of IAs and rupture state (aSAH)
Li et al., 2014 [73]	Plasma	Screening (20 UIA, 20 aSAH, 20 Ctrl), validation (93 IA, 50 Ctrl)	Prior to treatment	Agilent microarray/qPCR	UIA vs. Ctrl IA vs. Ctrl	miR-939, -126, -17, and 116 others let-7 family, miR-16, -25, and 13 others	Plasma miRNAs are significantly changed in patients with either aSAH or unruptured IAs.
Meeuwsen et al., 2017 [84]	Plasma	Discovery (15 aSAH of which 11 w/additional UIA, 15 Ctrl; Validation (15 aSAH, 15 UIA, 15 Ctrl)	Two years	Qiagen PCR array, qPCR	IA vs. Ctrl UIA vs. Ctrl aSAH vs. Ctrl	miR-200a-3p, -183-5p, Let-7b-5p let-7b-5p, miRNA-183-5p miR-200a-3p, -183-5p	Circulating miRNAs are able to discriminate between IA patients and controls.
Supriya et al., 2020 [80]	Plasma	Discovery (20 aSAH, 20 Ctrl), validate (68 aSAH, 90 Ctrl)	12 h post ictus	Exiqon PCR array, qPCR	aSAH vs. Ctrl	miR-15a-5p, -34a-5p, -374a-5p, -146a-5p, -376c-3p, -18b-5p, -24-3p, -27b-3p, and 69 other miRNAs	Eight miRNAs could serve as candidate biomarkers for IA rupture
Liao et al., 2020 [74]	Plasma exosome	30 UIA, 39 RIA (aSAH), 30 Ctrl	Within 7 days of RIA	NGS (8 aSAH, 4 UIA, 4 HC)/qPCR	UIA vs. Ctrl RIA vs. Ctrl RIA vs. UIA IA vs. Ctrl	miR-96-5p, -92a-1-5p, -17-3p and 26 others let-7a-2-3p, miR-1245a, -208b-3p, and 28 others miR-215-5p, -145-5p, -202-5p, and 118 others miR-29a-3p, and -145-5p	Circulating exosomal miRNAs can serve as biomarkers for IA development and progression
Sheng et al., 2018 [77]	Serum	129 aSAH, 40 Ctrl	Day 1, 3, 7, 14	qPCR	Poor prognosis	miR-502-5p	Higher miR-502-5p levels at day 7 were associated with a significantly high risk for poor outcome post-aSAH
Sheng et al., 2018 [78]	Serum	128 aSAH, 40 Ctrl	Day 1, 3, 7, 14	qPCR	Poor prognosis	miR-1297	Serum miR-1297 may help predict the prognosis in aSAH patients
Lai et al., 2017 [72]	Serum	60 aSAH, 13 Ctrl	Day 3	Exqion microarray (3 aSAH, 3 Ctrl), qPCR	aSAH vs. Ctrl aSAH severity and poor outcome	miR-502-5p, -1297 and miR-4320 and 11 others miR-502, and -1297	MiR-502-5p and miR-1297 are potential biomarkers for aSAH and associated with the prognosis.
Pulcrano-Nicolas et al., 2018 [76]	Whole blood	16 DCV+, 16 DCV−	Day 3	NGS, qPCR	DCV+ vs. DCV−	miR-3177-3p, and 441 others	Elevated miR-3177-3p levels in whole blood, which is accompanied with a decreased in LDHA. miR-3177-3p is a candidate marker for the risk of DCV
Lopes et al., 2018 [63]	Blood	14 DCV+, 13 DCV−, 6 Ctrl	Day 7–10	NGS	aSAH vs. Ctrl DCV+ vs. DCV−	let-7f-5p, miR-126-5p, -146a-5p, -17-5p, -451a, and 3 others; 15 novel miRNAs no significant DE miRNA	8 miRNAs were found to associate with aSAH, however, no miRNA was found to distinguish DCV. DE miRNAs related to MYC gene.
Korostynski et al., 2019 [62]	Whole blood	19 aSAH-acute phase, 20 aSAH-chronic phase, 20 Ctrl	Acute (≤72 hr), chronic (3–15 m)	NGS	acute vs. chronic	let-7 family, miR-142-3p, -145-5p, -155, -27a-3p, -223-3p, -451a and more than 100 others	Altered miRNAs are associated with cytokine-cytokine receptor interactions and inflammatory factors.

DCV: delayed cerebral vasospasm; DCI: delayed cerebral ischemia; Ctrl: control; aSAH: aneurysmal subarachnoid hemorrhage; IA: intracranial aneurysm; UIA: unruptured IA; RIA: ruptured IA; NGS: next-generation sequencing. * The authors gave different account of what biofluid (plasma, serum) was used. Based on the EDTA-tube being used for the collection, we believed the biofluid used was plasma.

**Table 3:** Pros and cons of commonly used biofluid miRNA detection platforms.

Detection Methods	Pros	Cons
RNA-Seq	Genome-wide coverage/discovery	Requires higher quantity of RNA/more expensive/lengthy procedure/complicated data analysis
Microarray	Genome-wide coverage	Requires higher quantity of RNA/low sensitivity and specificity/more expensive/lengthy procedure
nCounter	Fast turn-around time/sensitivity/able to use RNA samples of poor quality	No genome-wide coverage or discovery/less specific/requires special equipment
qPCR	Sensitivity/specificity/cost-effectiveness/easy to use/fast turn-around time	No genome-wide coverage or discovery

## Data Availability

Not applicable.
